# Liebig’s Soup for Invalids

**Published:** 1860-06

**Authors:** 


### Liebig's Soup for Invalids.

Take half pound of newly-killed beef or fowl, chop it fine, add 1⅛ lb. of distilled water, with four drops of pure muriatic acid, and 34 to 67 grains of common salt, and stir well together. After an hour the whole is to be thrown on a conical hair sieve and the fluid allowed to flow through without any pressure. The first thick portions which pass through are to be returned to the sieve, until the fluid run off quite clear. Half pound of distilled water is to be poured in, small portions at a time, on the flesh residue in the sieve.—There will be obtained in this way about a pound of fluid (cold extract of flesh), of a red colour, and having a pleasant taste of soup. The invalid is allowed to take it cold, a cupful at a time at pleasure. It must not be heated, as it becomes muddy by heat, and deposits a thick coagulum of albumen and colouring matter of blood. In soup, prepared in the usual way by boiling, all those contituents of flesh are wanting which are necessary for the formation of blood albumen; and the yolk of eggs which is added is poor in those substances, for it contains in all 82½ per cent. of water and fat, and only 17½ per cent. of a substance the same or very similar to albumen of egg. But whether it is equal in its power of nutrition to the albumen of flesh, is at least doubtful from the experiments of Magendie. Besides, the albumen of flesh, the new soup contains a certain quanty of colouring matter of blood, and with it a much larger quantity of the necessary iron for the formation of the blood corpuscles, and finally the muriatic acid to assist digestion. A great obstacle to the use of this soup in summer is, its liability to change in warm weather. The extraction of the flesh must consequently be made with very cold water, and in a cool place. Iced water, and external cooling with ice, completely remove this difficulty. But the most important point to be attended to, is to employ meat quite recently killed, and not several days old.—*Charleston Medical Review.*

